# Supplementary material for: Transition Metal Dichalcogenide Nanoflowers Rescue Immune Cells from the Cytotoxic Effects of Amyloid Aggregates
Source: bioRxiv. 2025 Oct 2:2025.10.02.680126. Preprint. [Version 1] doi: 10.1101/2025.10.02.680126 (PMC12622018; doi:10.1101/2025.10.02.680126)
Supplement: Supplement 1 [file media-1.pdf]

Transition Metal Dichalcogenide Nanoflowers Rescue Immune Cells from the Cytotoxic Effects of Amyloid Aggregates

Mikhail Matveyenka[1], Charles L. Mitchell[1], and Dmitry Kurouski[*1]

1. Department of Biochemistry and Biophysics, Texas A&M University, College Station, Texas 77843, United States

Email: dkurouski@tamu.edu

Supporting Information

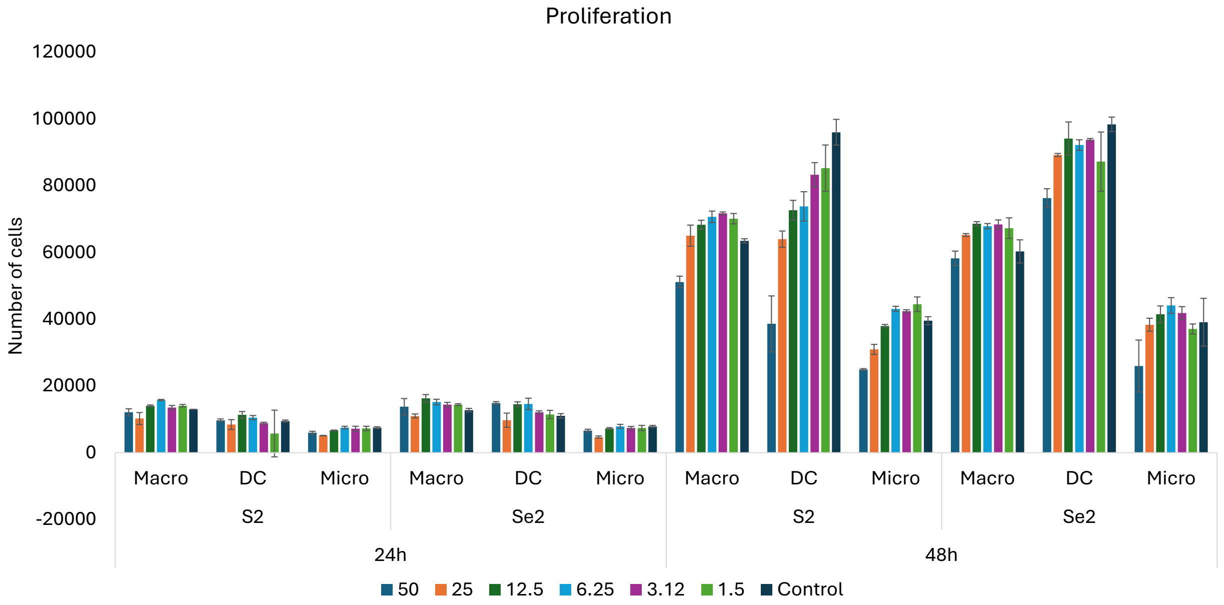

Figure S1. Proliferation of macrophages (macro), DC cells (DC) and microglia (micro) in the presence of 1.5-50 μM of MoS_2 (S2) and $MoSe_2$ (Se2).
